# Introspection, attention or awareness? The role of the frontal lobe in binocular rivalry

**DOI:** 10.3389/fnhum.2014.00527

**Published:** 2014-07-21

**Authors:** Natalia Zaretskaya, Marine Narinyan

**Affiliations:** Vision and Cognition Laboratory, Centre for Integrative Neuroscience, University of TübingenTübingen, Germany

**Keywords:** bistable perception, binocular rivalry, frontal lobe, consciousness, fMRI

Bistable stimuli are one of the most popular approaches to studying the neural mechanism of conscious visual perception. Such stimuli contain conflicting information, which the visual system cannot integrate into a unified percept. This causes the perceptual state of the observer to change every few seconds between the two interpretations while the physical stimulus remains the same. Binocular rivalry is an example of such perceptual phenomena with ambiguity achieved by presenting one image to one eye and a different image to the other eye. Perceptual changes during binocular rivalry are particularly vivid, and closely resemble a physical image exchange.

The study of neural mechanisms of bistable perception and binocular rivalry revealed the involvement of multiple areas across different levels of the visual hierarchy (Sterzer et al., 2009). While the activated stimulus-selective temporal lobe areas depend on the stimulus category that produces ambiguity, activity in the parietal and frontal lobe seems to be common to all stimuli and increases specifically during transition periods between the two percepts. The activation of the frontal lobe areas is particularly intriguing. It suggests that the mechanisms of conscious vision might involve typical higher-level frontal lobe functions such as attention, motivation and decision-making.

Previous studies of bistable perception relied on participants' explicit reports of their conscious state (most commonly button presses). Under certain conditions, however, perceptual states can be inferred objectively, without self-report. An example recent study by Frässle et al. (2014) took advantage of two ocular reflexes, optokinetic nystagmus and the pupillary light reflex, and designed binocular rivalry stimuli that selectively drive these reflexes. Simple eye tracking measures provided the authors with objective information about their subjects' perceptual changes over time.

The authors used fMRI to monitor brain activity when observers experienced either genuine binocular rivalry or a “replay” (a control condition which simulates rivalry-like perceptual alterations by physically switching the stimulus presented to both eyes). These conditions were performed while subjects either actively reported their perceptual state or just passively viewed the stimuli.

Such experimental design allowed Frässle et al. to make an important observation. Comparison of rivalry with replay in the passive viewing condition showed less activation in the frontal cortex compared to active report condition. The most prominent decrease was observed in the left superior and bilateral middle frontal gyri. According to the authors, decrease of frontal activity implies that frontal lobe areas play a role in introspection and motor response generation, but not in perceptual changes *per se*.

Frontal lobe takes about one third of the cortical volume, and is comprised of multiple architectonically and functionally distinct areas (Stuss, 2011). Given this diversity it is important to ask, which frontal lobe regions are typically active during perceptual transitions in bistable perception, and how they relate to the regions reported by Frässle et al. as showing reduced activation during passive viewing.

Previous studies comparing perceptual changes in rivalry with replay frequently reported activation in the inferior frontal cortex (IFC), middle frontal gyrus (MFG) and superior frontal gyrus (SFG) (Lumer et al., 1998; Zaretskaya et al., 2010; Knapen et al., 2011; Frässle et al., 2014), located mostly in the right hemisphere (Figure 1, green). Frässle et al. on the other hand, report decreased activity around the bilateral MFG and bilateral SFG only (Figure 3 and Table 2 in Frässle et al., 2014, Figure 1, red). Moreover, their right superior frontal activation is relatively far from the locations reported previously. Thus, the two activation maps in the frontal lobe overlap only partially, namely around the right MFG, and perhaps also the left SFG. Activity of other rivalry-related frontal nodes, in particular the right superior frontal and the right inferior frontal ones, did not decrease significantly. The latter areas may therefore continue to play an important role in perceptual changes even without active motor report.

**Figure 1:**
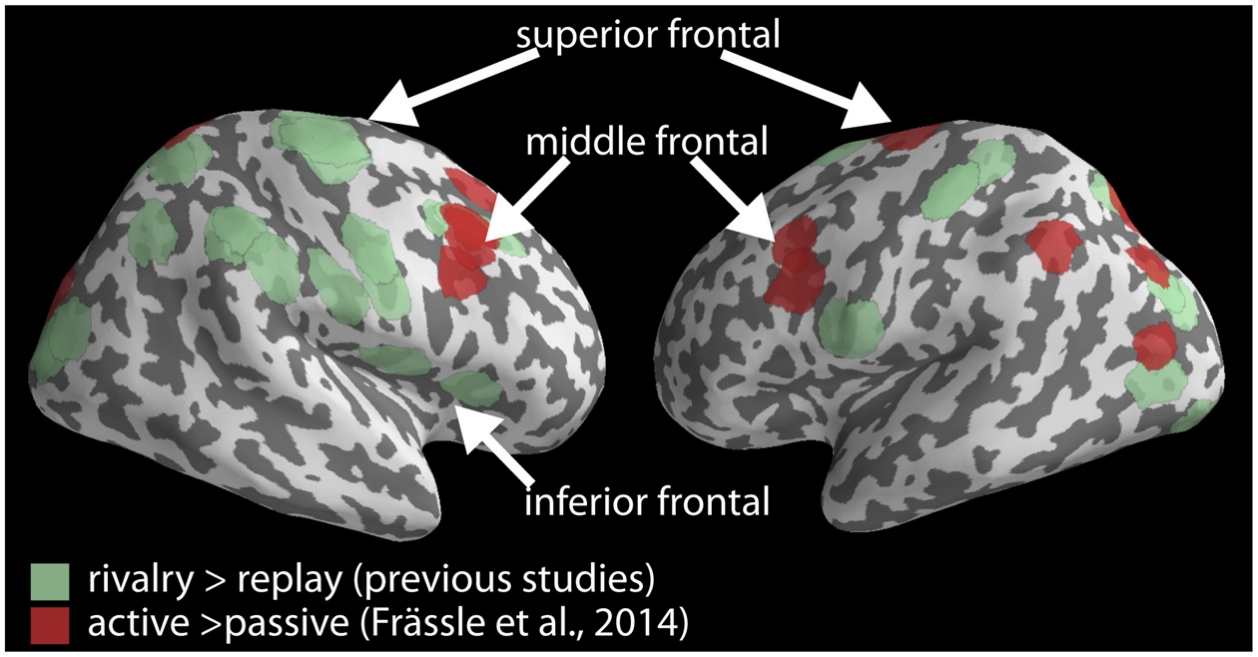
**Rivalry-related brain network**. Locations reported to be more active in rivalry compared to instantaneous replay based on observer's button presses in previous studies (green) are plotted together with locations that were less active during passive viewing than during the active report for the same comparison by Frässle et al. (red). Locations are plotted on a cortical surface reconstruction of the MNI brain using PySurfer (http://pysurfer.github.io). Coordinates of the locations in green are taken from the following studies: Lumer et al. (1998), Zaretskaya et al. (2010), Knapen et al. (2011), Frässle et al. (2014).

Besides, passive viewing involves not only less self-monitoring, but also less attention. Attention, in turn, is linked to increased activity in frontal, but also parietal and extrastriate areas. The reduction of activity at multiple non-frontal brain locations (Table 2 in Frässle et al., 2014, Figure 1) is in our view compatible with a more general attention effect. Difference in median dominance durations between active and passive conditions further support an attentional explanation. The authors report that, at least in the case of the pupil size-based analyses, median dominance durations were longer in the passive viewing condition. It is known that not only unreported but also unattended rivalry can lead to longer dominance durations (Paffen and Alais, 2011). In sum some of the fMRI effects are difficult to disentangle from attentional modulation.

Besides singling out the effects of self-monitoring from those of attention during passive viewing, it is important to dissociate the effects of self-monitoring and those of motor response. The ability to report one's conscious experience is not a prerequisite for conscious perception, but this may not hold for self-monitoring and introspection. Can we be sure that we are aware of something without reflecting about it? In fact, several approaches to consciousness define self-awareness as its essential component (Van Gulick, 2014). This raises some more philosophical questions about the nature of conscious experience that are beyond the scope of this review. In any case, we think that future studies could benefit from better segregation of different processes that accompany self-report and equating attentional demands between different conditions.

Almost two decades ago Crick and Koch (1995) proposed that extrastriate projections to the frontal cortex are necessary for conscious perception, thus putting the frontal lobes front and center in the study of neural correlates of consciousness. Indeed, experimental findings on bistable perception over the years supported the important role of frontal areas in generating perceptual changes (e.g., Sterzer and Kleinschmidt, 2007; Weilnhammer et al., 2013). The study of Frässle et al. does point to a possible confound of active motor report in perception-related activity of some frontal lobe sites. However, it does not rule out the possibly important role of other frontal lobe areas in generating conscious visual experience.

## Conflict of interest statement

The authors declare that the research was conducted in the absence of any commercial or financial relationships that could be construed as a potential conflict of interest.
